# Efficacy of Intravitreal Bevacizumab in Treatment of Proliferative Type 2 Idiopathic Juxtafoveal Telangiectasia

**DOI:** 10.4274/tjo.04874

**Published:** 2017-06-01

**Authors:** Ökkeş Baz, İhsan Yılmaz, Cengiz Alagöz, Ali Demircan, İrfan Perente, Abdullah Özkaya, Muhittin Taşkapılı

**Affiliations:** 1 Prof. Dr. Reşat N. Belger Beyoğlu Eye Training and Research Hospital, Ophthalmology Clinic, İstanbul, Turkey

**Keywords:** Juxtafoveal telangiectasia, subretinal neovascularization, Bevacizumab

## Abstract

**Objectives:**

To evaluate the effectiveness of intravitreal bevacizumab (IVB) in patients with subretinal neovascularization secondary to type 2 juxtafoveal telangiectasia.

**Materials and Methods:**

Ten eyes of 10 patients were included in this retrospective study. All cases were treated with IVB (1.25 mg bevacizumab). Visual acuity and slit-lamp anterior and posterior segment examinations were performed at each visit. Central macular thickness (CMT) and intraretinal/subretinal fluid were evaluated via spectral domain optical coherence tomography (OCT). Loss of a line in visual acuity chart and presence of fluid on OCT were defined as criteria for repeated treatment.

**Results:**

The mean age of patients was 66.0±7.0 years (56-75). The mean follow-up time was 54.7±16.0 month (24-72). The mean BCVA was 0.62±0.35 (0.00-1.00) logMAR at baseline and 0.54±0.35 (0.00-1.00) logMAR at final exam (p=0.03). The mean CMT was 251±25.4 µm at baseline and 239±39.3 µm at final exam (p=0.01). Patients received an average of 1.7±1.0 IVB injections during follow-up. At baseline, all cases had intraretinal/subretinal fluid. There was no fluid at final examination of all cases.

**Conclusion:**

IVB treatment may be effective in the treatment of subretinal neovascularization secondary to type 2 juxtafoveal telangiectasia.

## INTRODUCTION

Retinal telangiectasia is generally idiopathic, but may also accompany various inflammatory and vascular pathologies.^1,2^ It was first described in 1956 by Reese.^3^ The current classification system for retinal telangiectasia was developed by Yannuzzi et al.^2^ based on optical coherence tomography (OCT) findings. According to this system, juxtafoveal telangiectasia (JFT) is divided into two groups. Type 1 JFT features cystic macular edema, retinal thickening, and exudations, while type 2 JFT is characterized by perifoveal telangiectasia.^2^

Type 2 JFT is more common. The condition results in reduced visual acuity and metamorphopsia after an average age of 50. It nearly always manifests bilaterally (98%), and affects males and females equally.^1,2,4^ The prevalence of type 2 JFT was reported as 0.1% in the Beaver Dam study.^5^ According to the Yannuzzi classification, type 2 JFT consists of 5 stages. Findings associated with each stage are: occult telangiectatic vessels in stage 1; loss of retinal transparency in stage 2; dilated right-angle venules in stage 3; pigment hyperplasia into the retina in stage 4; and choroidal neovascularization in stage 5. The first 4 stages are referred to as the nonproliferative stage, and stage 5 as the proliferative stage.^2^

Laser photocoagulation, photodynamic therapy (PDT), intravitreal triamcinolone injection (IVTA), carbonic anhydrase inhibitors, and intravitreal anti-vascular endothelial growth factor (VEGF) injections are used in the treatment of nonproliferative type 2 JFT.6 Treatment options for subretinal neovascularization (SRNV) associated with type 2 JFT include PDT, IVTA, and surgical interventions. The main disadvantage of PDT is subsequent retinal pigment epithelium atrophy. IVTA therapy poses a risk of cataract and glaucoma development and surgical removal of SRNV is difficult, leading to the search for new therapeutic options.^7,8^ Although the pathogenesis of SRNV related to type 2 JFT is not completely understood, it is known that VEGF plays an important role. Anti-VEGF therapy has been shown to reduce retinal edema and fluid leakage.^9,10,11,12,13,14^

The purpose of this study was to investigate the long-term anatomic and functional outcomes of intravitreal bevacizumab (IVB) injection in the treatment of SRNV secondary to type 2 JFT.

## MATERIALS AND METHODS

### Participants

We retrospectively analyzed the medical records of patients followed for type 2 JFT in our hospital from January 2009 to January 2014. Patients treated with IVB for SRVN secondary to proliferative type 2 JFT were included in the study. Patients with history of vitreoretinal surgery or other retinal disease were not included.

### Ophthalmologic Examination

All subjects underwent a standard ophthalmologic examination prior to treatment. Visual acuity measurements were obtained in photopic conditions (85 candela/m^2^) using the Bailey-Lovie chart at a distance of 4 meters. Anterior segment and fundus were evaluated by slit-lamp examination and intraocular pressure was measured by Goldmann applanation tonometry. Spectral domain OCT (SD-OCT) images were acquired at each visit using the Spectralis (Heidelberg Engineering, Heidelberg, Germany) instrument. The same instrument was used for fundus fluorescein angiography (FFA). FFA was conducted at time of diagnosis and at follow-up visits if patients exhibited vision loss and other examination methods were unable to reveal its cause. Follow-up examinations were done at 1, 3, 6, and 12 months, and at 6-month intervals thereafter.

### Treatment Protocol

Patients were informed about the side effects and risks associated with IVB (Avastin; Genentech Inc, San Francisco, CA, USA) therapy and informed consent forms were obtained. All injections were done in sterile conditions. Before injection, the eyelids were cleaned with 10% povidone iodine (Betadine; Purdue Pharma, Stamford, CT, USA) and the conjunctival sac with 5% povidone iodine. After placing a sterile cover, IVB (1.25 mg/0.05 mL) was injected using a 30-gauge needle inserted 3.5 mm from the limbus in pseudophakic patients and 4 mm from the limbus in phakic patients. Patients used topical 0.5% moxifloxacin ophthalmic solution (Vigamox^®^, Alcon Laboratories Inc., Fort Worth, TX, USA) for 1 week after injection. Patients were examined monthly after the first injection and repeated injections were administered if necessary. Loss of a line or more in visual acuity and the presence of subretinal hemorrhage, intraretinal cyst, and/or subretinal fluid were defined as criteria for repeated treatment.

### Statistical Analysis

Data were analyzed using SPSS version 22.0 (SPSS Inc., Chicago, IL, USA). Monthly changes in visual acuity and central macular thickness (CMT) values were compared using a paired-samples t-test. P values ≤0.05 were considered statistically significant.

## RESULTS

Ten eyes of 10 patients (7 female, 3 male) were included in the study. Mean age of the patients was 66±70 (56-75) years. Mean follow-up time was 54.7±16 (24-72) months. Twenty percent of the patients had diabetes mellitus. Patient’s fellow eyes had nonproliferative type 2 JFT. Intraretinal crystalline accumulation was observed in 3 patients (30%). Demographic characteristics of the patients are shown in Table 1.

**Figure 1:**
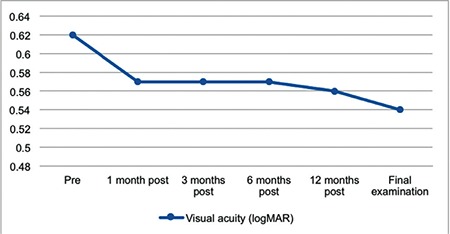
Visual acuity changes from before treatment (pre) to after treatment (post)

Mean best corrected visual acuity (BCVA) before treatment was 0.62±0.35 (0.00-1.00) logMAR. Post-treatment mean BCVA values were 0.57±0.35 (0.00-1.00) logMAR at 3 months (p=0.10), 0.56±0.36 (0.00-1.00) logMAR at 12 months (p=0.06), and 0.54±0.35 (0.00-1.00) logMAR at final examination (p=0.03). Only the difference between baseline and final BCVA was statistically significant (Figure 1). BCVA improved by 1 line in 2 eyes and 2 lines in 3 eyes. Visual acuity remained at the same level in 5 eyes. Changes in BCVA are shown in Figure 1.

Mean CMT before treatment was 251±25.4 (197-283) µm. Post-treatment CMT values were 245±27 (186-280) at 3 months (p=0.02), 245±40 (168-222) µm at 12 months (p=0.30), and 239±39.3 (160-310) µm at final examination (p=0.01). Changes in CMT were statistically significant at 3 months and final examination. All patients had intraretinal and/or subretinal fluid prior to injection. In the final examinations, intra- and/or subretinal fluid was not detected in any of the patients on SD-OCT.

The patients received an average of 1.7±1.05 IVB injections. IVB injections were administered once to 6 eyes, twice to 2 eyes, 3 times to 1 eye, and 4 times to 1 eye. None of the patients experienced serious injection-related complications such as retinal detachment, endophthalmitis, or vitreous hemorrhage.

## DISCUSSION

**Figure 2:**
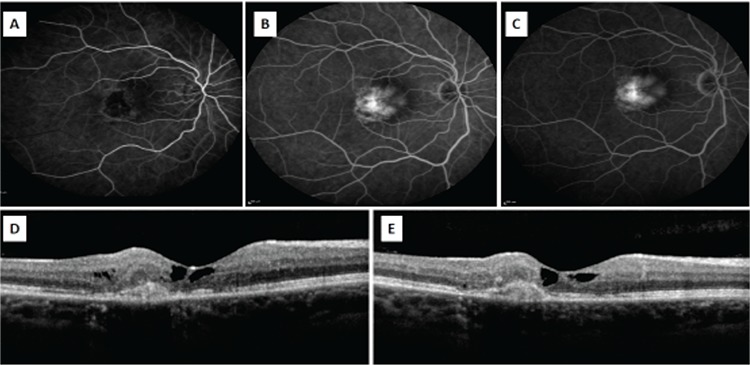
A-C) Fundus fluorescein angiography in a patient with type 2 juxtafoveal telangiectasia shows hyperfluorescence due to subretinal neovascularization (SRNV) beginning in the early phase and increasing in the later phases. D) Optical coherence tomography reveals the internal limiting membrane, foveal atrophy, and SRNV and intraretinal fluid temporal of the fovea. E) Regression of the intraretinal fluid is observed after intravitreal injection

**Figure 3:**
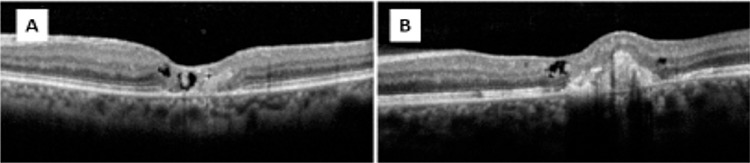
A) Optical coherence tomography (OCT) images of a patient with type 2 juxtafoveal telangiectasia reveals internal limiting membrane coverage and foveal atrophy. B) Subretinal neovascularization formation was noted on follow-up OCT images of the same patient taken three years later

The mechanism by which SRNV develops in type 2 JFT is not clear. SRNV usually begins intraretinally and progresses to the subretinal surface, but does not become widespread (Figures 2 and 3). However, if untreated the prognosis of SRVN is poor.15 One study that followed 26 untreated eyes for an average of 107 months found that visual acuity was 20/200 or worse in 81% of the eyes.^16^ Various studies have reported that bevacizumab is safe and effective in the treatment of SRNV secondary to myopia and age-related macular degeneration.^17,18^

In the present study, we observed no reduction in visual acuity in the 10 eyes with type 2 JFT treated with IVB injection. Visual acuity remained stable in 5 eyes (50%) and increased by 1 or more lines in 5 eyes (50%). None of the eyes had sub-/intraretinal fluid and there was a significant decrease in CMT in OCT examination done at the end of follow-up.

In a similar study, Mandal et al.^19^ administered a single dose of IVB to 6 eyes with JFT-associated SRNV and reported visual acuity improvement of 2 or more lines in 5 eyes (83%) and no change in 1 eye at follow-up examination 4 months after injection. Narayanan et al.^20^ administered IVB to 12 eyes and intravitreal ranibizumab injections to 4 eyes of 16 patients with JFT-associated SRNV and observed a significant increase in visual acuity after a follow-up period of 12 months. They reported the average number of annual injections as 1.9. In a study by Jorge et al.,^21^ an eye with SRNV secondary to type 2 JFT was treated with IVB and at 24 weeks after treatment, visual acuity increased from 20/40 to 20/20 and the subretinal fluid resolved on OCT. Roller et al.^22^ reported a 1.1 line increase in visual acuity at the end of 17 months of follow-up after IVB treatment of 9 type 2 JFT patients.

Recurrence is rare after the first injection when treating SRNV secondary to type 2 JFT. Karagiannis et al.^23^ administered 3 monthly doses of ranibizumab to a patient and reported an increase in visual acuity from 0.05 to 0.3 and no recurrence during 12 months of follow-up. We performed an average of 1.7 injections over the course of 5 years of follow-up in our patients, and 6 eyes were successfully treated with a single injection. Our injection number was similar to those reported in other studies. Over the course of long-term follow-up, fewer injections are required in the treatment of type 2 JFT-associated SRNV when compared to age-related macular degeneration. This result demonstrates that there is little need for anti-VEGF therapy when managing SRNV secondary to type 2 JFT.

A strength of our study compared to previous studies was our long follow-up time and relatively larger number of cases. Limitations of our study include its retrospective design and lack of a control group.

## CONCLUSION

In summary, IVB is effective in stabilizing visual acuity and slowing the progression of SRNV in the treatment of SRNV secondary to type 2 JFT. Controlled, randomized studies with larger patient numbers are needed to evaluate the anatomic and functional outcomes of IVB therapy in type 2 JFT.

## Figures and Tables

**Table 1:**
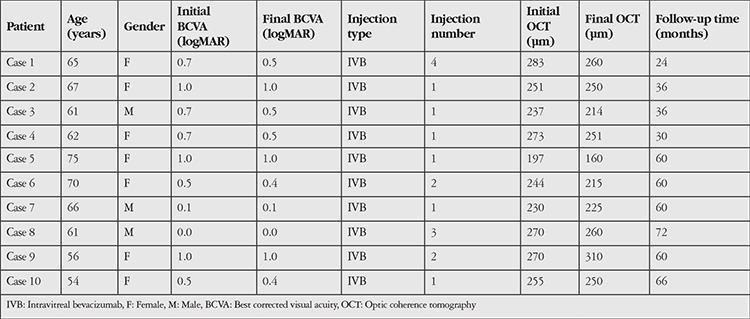
Patients’ demographic and treatment characteristics
